# Timing of Confirmatory Trials for Drugs Granted Accelerated Approval Based on Surrogate Measures From 2012 to 2021

**DOI:** 10.1001/jamahealthforum.2023.0217

**Published:** 2023-03-31

**Authors:** Anjali D. Deshmukh, Aaron S. Kesselheim, Benjamin N. Rome

**Affiliations:** 1 Program on Regulation, Therapeutics, and Law (PORTAL), Division of Pharmacoepidemiology and Pharmacoeconomics, Department of Medicine, Brigham and Women’s Hospital, Boston, Massachusetts; 2 Georgia State University College of Law, Atlanta, Georgia; 3 Harvard Medical School, Boston, Massachusetts

## Abstract

This cross-sectional study systematically characterizes the frequency of delayed confirmatory trials relative to deadlines set by the US Food and Drug Administration.

## Introduction

The US Food and Drug Administration (FDA) accelerated approval program expedites regulatory approval of certain medications for patients with serious illness using efficacy evidence from surrogate markers that are reasonably likely to predict clinical benefits. As required by law, drug manufacturers agree to evaluate clinical benefits in a follow-up trial. The FDA sets a time frame for completion, but confirmatory trials are sometimes delayed.^1,2,3^ We sought to systematically characterize the frequency of delayed confirmatory trials relative to deadlines set by the FDA.

## Methods

In this cross-sectional study, we used public FDA data to identify follow-up trial requirements and the deadline agreed on between FDA and manufacturer for drugs granted accelerated approval from January 1, 2012, to July 31, 2021. We compared the time granted by characteristic (therapeutic area and small molecule vs biologic) using Wilcoxon rank sum tests at a significance level of *P* < .05. Trials were categorized as late if the requirement was fulfilled (met or released by the FDA) or the indication withdrawn by the manufacturer after the agreed on deadline, or if the requirement remained unfulfilled after the deadline, as of September 15, 2021. We also identified manufacturer-reported delays among incomplete requirements as of September 15, 2021. This study followed the STROBE reporting guideline. Per the Common Rule (45 CFR §46), institutional review board review and approval were not sought because this study does not constitute human participant research. Results were analyzed in Excel (Microsoft) and SAS, version 9.4 (SAS Institute Inc).

## Results

We included 177 follow-up trial requirements among 140 new indications (range, 0-4 requirements per indication). The median time allowed to complete confirmatory trials was 3.5 years from the date of approval (IQR, 1.8-5.8 years), with significant variation by therapeutic area (Table). The time granted for completion remained steady over the study period and did not vary between biologics and small molecule drugs (median 3.3 [IQR, 1.8-5.4] years vs 3.8 [IQR, 1.8-6.1] years; *P* = .63).

**Table.** Characteristics of Accelerated Approval Confirmatory Trial Requirements

Drug characteristic	No. clinical trial requirements^a^	Median time between approval and deadline (IQR), y	No. trials completed or due by September 15, 2021	Late trials, No. (%)
All	177	3.5 (1.8-5.8)	100	54 (54)
Therapeutic area^b^				
Genetic disease	8	8.7 (4.6-9.3)	1	1 (100)
Infectious disease	6	4.2 (2.0-8.0)	0	0
Neurology	2	8.0 (7.6-8.4)	1	1 (100)
Hematology	15	2.5 (1.1-4.0)	14	13 (93)
Autoimmune disease	4	6.9 (6.5-6.9)	0	0
Cancer	142	3.5 (1.8-5.6)	84	39 (46)
Drug type				
Small molecule	110	3.7 (1.8-6.1)	65	40 (62)
Biologic	67	3.3 (1.8-5.4)	35	14 (40)

^a^ We identified accelerated approvals from January 1, 2012 and July 31, 2021 using the CDER Drug and Biologic Accelerated Approval Based on Surrogate Endpoint (https://www.fda.gov/drugs/nda-and-bla-approvals/accelerated-approvals). Postmarket trial requirements and the deadlines for each requirement were identified from correspondence on Drugs@FDA and ranged from 0.1-14.0 years after approval.

^b^ Therapeutic area was categorized by the authors. Treatment categories with fewer than 5 indications (autoimmune disease and neurology) were combined for analysis. There was a significant variation in time granted by therapeutic area.

One-hundred trials (57%) were completed or due before September 15, 2021, of which 54 (54%) were late (Figure). An additional 14 studies (8%) were reported to the FDA as delayed by the manufacturer before the due date. Trials for nononcologic indications (93% [15 of 16]) were more likely to be late than those for oncologic indications (46% [39 of 84]). Trials for small molecule drugs (62% [40 of 65]) were more likely to be late than those for biologics (40% [14 of 35]).

**Figure.**
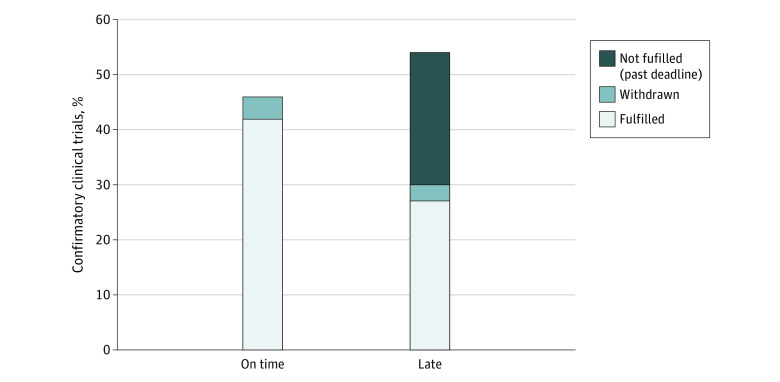
Timeliness of Required US Food and Drug Administration Confirmatory Clinical Trials The timeliness of the 100 clinical trial requirements that were completed or due before September 15, 2021. Not shown are an additional 77 unfulfilled requirements with deadlines after September 15, 2021.

Of the 54 late requirements, 27 were fulfilled by September 15, 2021, a median 1.0 year (IQR, 0.7-1.9 years) after the deadline and 3 were withdrawn a median of 1.4 years (range, 0.9-3.3 years) after the deadline. Twenty-four incomplete late requirements were a median of 1.8 years (IQR, 0.6-2.2 years) past their deadlines as of September 15, 2021.

## Discussion

Accelerated approval expedites the marketing of medications based on uncertain efficacy evidence, but the process depends on timely follow-up trials. We found that more than half of so-called confirmatory studies were not completed in the agreed-on time. In contrast with a recent Office of the Inspector General Report^4^ of incomplete confirmatory trials, our study includes late completed trials and manufacturer-reported delays. Limitations include shorter follow-up for more recent accelerated approvals and inability to identify reasons for trial delays.

Incomplete confirmatory clinical trials harm patients who are prescribed expensive drugs despite uncertain clinical benefits.^5^ However, drug manufacturers face few consequences for delays. The Consolidated Appropriations Act for 2023^6^ included accelerated approval reforms, such as granting the FDA greater authority to ensure confirmatory trials are under way before approval, mandating progress reports every 6 months by manufacturers, and clarifying procedures for withdrawal if follow-up trials do not find clinical benefit. It will be important to monitor whether these changes lead to fewer delays or whether additional authority is needed to assure that confirmatory trials are completed in a timely manner for the benefit of patients.

## Notes

Supplement.
Data Sharing Statement
