# Supplementary material for: Timing of Confirmatory Trials for Drugs Granted Accelerated Approval Based on Surrogate Measures From 2012 to 2021
Source: JAMA Health Forum. 2023 Mar 31;4(3):e230217. doi: 10.1001/jamahealthforum.2023.0217 (PMC10066454; doi:10.1001/jamahealthforum.2023.0217)
Supplement: Supplement. — Data Sharing Statement [file jamahealthforum-e230217-s001.pdf]

Data Sharing Statement

Deshmukh. Timing of Confirmatory Trials for Drugs Granted Accelerated Approval Based on Surrogate Measures From 2012 to 2021. *JAMA Health Forum*. Published March 31, 2023. doi:10.1001/jamahealthforum.2023.0217

Data
Data available: Yes
Data types: Data (not involving human participants)
How to access data: Data are all from public FDA sources.
When available: With publication

Supporting Documents
Document types: None

Additional Information
Who can access the data: Anyone requesting the data.
Types of analyses: Any purpose.
Mechanisms of data availability: From public FDA sources.
